# First‐line nivolumab plus chemotherapy vs chemotherapy in patients with advanced gastric, gastroesophageal junction and esophageal adenocarcinoma: CheckMate 649 Chinese subgroup analysis

**DOI:** 10.1002/ijc.34296

**Published:** 2022-10-31

**Authors:** Tianshu Liu, Yuxian Bai, Xiaoyan Lin, Wei Li, Jufeng Wang, Xiaochun Zhang, Hongming Pan, Chunmei Bai, Li Bai, Ying Cheng, Jingdong Zhang, Haijun Zhong, Yi Ba, Wenwei Hu, Ruihua Xu, Weijian Guo, Shukui Qin, Nong Yang, Jianwei Lu, Kohei Shitara, Ming Lei, Mingshun Li, Nicole Bao, Tian Chen, Lin Shen

**Affiliations:** ^1^ Zhongshan Hospital Fudan University Shanghai China; ^2^ Herbin Medical University Heilongjiang China; ^3^ Fujian Medical University Union Hospital Fuzhou China; ^4^ The First Hospital of Jilin University Changchun China; ^5^ The Affiliated Cancer Hospital of Zhengzhou University Henan Cancer Hospital Zhengzhou China; ^6^ The Affiliated Hospital of Qingdao University Qingdao China; ^7^ Sir Run Run Shaw Hospital Hangzhou China; ^8^ Peking Union Medical College Hospital Beijing China; ^9^ China P.L.A. General Hospital (301 Hospital) Beijing China; ^10^ Jilin Cancer Hospital Changchun China; ^11^ Liaoning Cancer Hospital and Institute Shenyang China; ^12^ Zhejiang Cancer Hospital Hangzhou China; ^13^ Tianjin Medical University Cancer Institute and Hospital Tianjin China; ^14^ The First People's Hospital of Changzhou Changzhou China; ^15^ Medical Oncology Cancer Center Sun Yat‐Sen University Guangzhou China; ^16^ Fudan University Shanghai Cancer Center Shanghai China; ^17^ Eastern Theater General Hospital QinHuai District Medical Area China; ^18^ Hunan Cancer Hospital Changsha Shi China; ^19^ Jiangsu Cancer Hospital Nanjing China; ^20^ National Cancer Center Hospital East Kashiwa Japan; ^21^ Bristol Myers Squibb Princeton New Jersey USA; ^22^ Department of Gastrointestinal Oncology Key Laboratory of Carcinogenesis and Translational Research (Ministry of Education/Beijing) Peking University Cancer Hospital and Institute Beijing China

**Keywords:** Asian Continental Ancestry Group, gastrointestinal neoplasms, immunotherapy, nivolumab, stomach neoplasms

## Abstract

First‐line chemotherapy for advanced/metastatic human epidermal growth factor receptor 2 (HER2)‐negative gastric/gastroesophageal junction cancer (GC/GEJC) has poor median overall survival (OS; <1 year). We report efficacy and safety results from Chinese patients in the phase III global CheckMate 649 study of nivolumab plus chemotherapy vs chemotherapy for the first‐line treatment of GC/GEJC/esophageal adenocarcinoma (EAC). Chinese patients with previously untreated advanced or metastatic GC/GEJC/EAC were randomized to receive nivolumab (360 mg Q3W or 240 mg Q2W) plus chemotherapy (XELOX [capecitabine and oxaliplatin] Q3W or FOLFOX [oxaliplatin, leucovorin and 5‐fluorouracil] Q2W), nivolumab plus ipilimumab (not reported) or chemotherapy alone. OS, blinded independent central review‐assessed progression‐free survival (PFS), objective response rate (ORR), duration of response (DOR) and safety are reported. Of 1581 patients enrolled and randomized, 208 were Chinese. In these patients, nivolumab plus chemotherapy resulted in clinically meaningful improvement in median OS (14.3 vs 10.2 months; HR 0.61 [95% CI: 0.44‐0.85]), median PFS (8.3 vs 5.6 months; HR 0.57 [95% CI: 0.40‐0.80]), ORR (66% vs 45%) and median DOR (12.2 vs 5.6 months) vs chemotherapy, respectively. The safety profile was acceptable, with no new safety signals observed. Consistent with results from the global primary analysis of CheckMate 649, nivolumab plus chemotherapy demonstrated a clinically meaningful improvement in OS and PFS and higher response rate vs chemotherapy and an acceptable safety profile in Chinese patients. Nivolumab plus chemotherapy represents a new standard first‐line treatment for Chinese patients with non‐HER2‐positive advanced GC/GEJC/EAC.

## Graphical abstract

What's new?

First‐line nivolumab plus chemotherapy demonstrated superior overall survival versus chemotherapy and an acceptable safety profile in advanced gastric/gastroesophageal junction/esophageal adenocarcinoma in the global CheckMate 649 trial. Chinese patients have high unmet need in this disease setting, and in this analysis, nivolumab plus chemotherapy demonstrated clinically meaningful improvement in efficacy versus chemotherapy with an acceptable safety profile in Chinese patients from CheckMate 649.

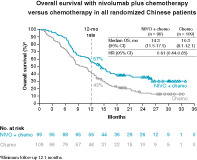

**Abbreviations**
AE: adverse event
BICR: blinded independent central review
CI: confidence interval
CPS: combined positive score
DOR: duration of response
EAC: esophageal adenocarcinoma
ECOG PS: Eastern Cooperative Oncology Group performance status
FOLFOX: oxaliplatin, leucovorin and 5‐fluorouracil
GC: gastric cancer
GEJC: gastroesophageal junction cancer
HER2: human epidermal growth factor receptor 2
HR: hazard ratio
LLN: lower limit of normal
MSI‐H: microsatellite instability‐high
MSS: microsatellite stable
ORR: objective response rate
OS: overall survival
PD‐1: programmed death‐1
PD‐L1: programmed death ligand 1
PFS: progression‐free survival
Q2W: every 2 weeks
Q3W: every 3 weeks
RECIST: Response Evaluation Criteria in Solid Tumors
TRAE: treatment‐related adverse event
XELOX: capecitabine and oxaliplatin

## 1 INTRODUCTION

Gastric cancer (GC) is the fifth most commonly diagnosed cancer and the fourth leading cause of cancer‐related death worldwide.^1^ GC, which is often defined to include gastroesophageal junction cancer (GEJC), is especially prevalent in China where it is the second most common cancer and the third leading cause of cancer‐related death annually.^2^ The most common histological type of GC/GEJC is adenocarcinoma, which accounts for more than 90% of GC and GEJC cases^3^ and approximately 15% of esophageal cancers worldwide.^4^ Esophageal adenocarcinoma (EAC) and GC/GEJC have comparable molecular profiles and follow similar treatment guidelines in most countries, including China.^3^, ^5^, ^6^, ^7^, ^8^, ^9^, ^10^, ^11^, ^12^ Until recently, standard first‐line treatment for unresectable advanced or metastatic human epidermal growth factor receptor 2 (HER2)‐negative gastric and gastroesophageal junction adenocarcinoma has been fluoropyrimidine plus platinum‐based chemotherapy, which results in poor median overall survival (OS) of <1 year.^5^, ^9^, ^10^, ^13^, ^14^, ^15^, ^16^, ^17^

CheckMate 649 is the largest, randomized phase III study of first‐line PD‐1 inhibitor‐based therapies in patients with unresectable advanced or metastatic, nonHER2‐positive GC/GEJC/EAC. In the primary analysis, nivolumab plus chemotherapy showed superior OS benefit vs chemotherapy alone in patients whose tumors expressed programmed death (PD) ligand 1 (L1) combined positive score (CPS) ≥5 (hazard ratio [HR] 0.71, *P* < .0001) and PD‐L1 CPS ≥1 (HR 0.77, *P* < .0001) and in all randomized patients (HR 0.80, *P* = .0002).^18^ Nivolumab plus chemotherapy also provided clinically meaningful progression‐free survival (PFS) benefit compared with chemotherapy in patients with PD‐L1 CPS ≥5 (HR 0.68, *P* < .0001) and PD‐L1 CPS ≥1 (HR 0.74) and in all randomized patients (HR 0.77). Furthermore, patients in the nivolumab plus chemotherapy group had a higher objective response rate (ORR), with more durable responses and more complete responses compared with the chemotherapy group. Nivolumab plus chemotherapy had an acceptable safety profile, and no new safety signals were identified.^18^

A meta‐analysis of immune checkpoint inhibitor monotherapy for the treatment of GC/GEJC indicated that Asian patients as a subgroup had greater OS benefit than non‐Asian patients; this benefit has also been observed in other tumor types.^19^, ^20^ Potential factors that could influence survival benefit include differences in immune cells expressing pro‐inflammatory neutrophil or macrophage markers such as CD66b and CD68, T cell markers such as CD3, CD8 and CD45R0, and immune‐suppressive markers such as FOXP3, which were associated with geographic locality‐specific prognosis of gastric adenocarcinomas in Asian vs non‐Asian patients.^21^ Additionally, differences exist in disease characteristics in Chinese patients such as primary tumor location (more frequently lower GC and less frequently middle GC), histology (less frequently diffuse) and frequency of gastrectomy (more frequently proximal gastrectomy and less frequently total gastrectomy) vs patients in other regions.^22^ Due to these known differences in clinical factors in Chinese patients, a population significantly affected by GC and GEJC, we conducted a preplanned analysis of the efficacy and safety of nivolumab plus chemotherapy vs chemotherapy alone in Chinese patients enrolled in the global CheckMate 649 study.

## 2 METHODS

### 2.1 Study design

This multicenter, open‐label, randomized phase III study enrolled patients at 175 hospitals and cancer centers in 29 countries, including 22 centers in China. Patients were initially randomized to receive nivolumab plus ipilimumab or chemotherapy alone at a 1:1 ratio. Amendments to the protocol resulted in randomization at a 1:1:1 ratio to nivolumab plus chemotherapy (XELOX [capecitabine and oxaliplatin] or FOLFOX [oxaliplatin, leucovorin and 5‐fluorouracil]), nivolumab plus ipilimumab or chemotherapy alone; subsequently, the nivolumab plus ipilimumab treatment group was closed, and patients were randomized in a 1:1 manner to treatment with nivolumab plus chemotherapy or chemotherapy alone. Patients were randomized with interactive web response technology in block sizes of six. Once informed consent was obtained from the patient, they were enrolled and assigned to treatment. A treatment allocation list was generated by the sponsor. Web registration was implemented by a third party, and the sequence of assignment was concealed until treatment allocation was complete. Investigators were not blinded to treatment allocation since the trial was open label.

Stratification factors were tumor cell PD‐L1 expression (≥1% vs <1% including indeterminate), Eastern Cooperative Oncology Group performance status (ECOG PS; 0 vs 1), chemotherapy type (XELOX vs FOLFOX) and region (Asia vs United States and Canada vs rest of world). All patients in the Chinese subgroup analysis were Asian. Additional amendments to the protocol resulted in patients with PD‐L1 CPS ≥5 being the primary population although patients continued to be enrolled regardless of PD‐L1 expression.

### 2.2 Patients

Detailed methods were previously described.^18^ Eligible patients had unresectable advanced or metastatic GC, GEJC or EAC and had not received prior systemic therapy. Prior adjuvant or neoadjuvant chemotherapy, radiotherapy and/or chemoradiotherapy was permitted if the last administration of the prior regimen was given at least 6 months before randomization. Other inclusion criteria were 18 years of age or older, histologically confirmed predominant adenocarcinoma histology with measurable (at least one lesion) or evaluable disease per Response Evaluation Criteria in Solid Tumors (RECIST) version 1.1, an ECOG PS of 0 or 1 and an available tumor sample with evaluable PD‐L1 results. Patients with known HER2‐positive status; untreated central nervous system metastases; ascites that could not be controlled with appropriate interventions; peripheral neuropathy of grade > 1; active, known or suspected autoimmune disease; positive test results for hepatitis B or C virus; or known history of positive test results for HIV or known AIDS and patients who had a live/attenuated vaccine within 30 days of first treatment, or treatment with botanical preparations (eg, herbal supplements or traditional Chinese medicines) intended to treat the disease under study within 2 weeks prior to randomization/treatment were excluded.

### 2.3 Treatments

Patients received nivolumab plus investigator's choice of XELOX (360 mg nivolumab day 1, 130 mg/m^2^ oxaliplatin day 1 and 1000 mg/m^2^ capecitabine twice a day days 1‐14 every 3 weeks) or FOLFOX (240 mg nivolumab day 1, 85 mg/m^2^ oxaliplatin day 1, 400 mg/m^2^ leucovorin day 1 and 400 mg/m^2^ 5‐fluorouracil day 1 and 1200 mg/m^2^ 5‐fluorouracil on days 1 and 2 every 2 weeks) or either chemotherapy regimen alone. All agents were given intravenously with the exception of capecitabine, which was given orally. Nivolumab and chemotherapy were provided by the study sponsor except for countries where local regulations allowed commercial procurement of chemotherapy. Chemotherapy was administered per local standard of care, and dose reductions were permitted per local standards to manage treatment‐related toxicity. Dose reductions were not permitted for nivolumab. Treatment was administered until disease progression, unacceptable toxicity, patient withdrawal of consent or the maximum treatment duration (24 months) of nivolumab per protocol. In the nivolumab plus chemotherapy group, treatment with nivolumab monotherapy or with chemotherapy alone was permitted if the other component was discontinued. In both groups, if individual components of chemotherapy were discontinued, treatment could continue with the remaining agents. Treatment with nivolumab beyond progression was permitted based on investigator judgment.

### 2.4 Endpoints

The dual primary endpoints for nivolumab plus chemotherapy vs chemotherapy in the global population were OS and PFS per blinded independent central review (BICR) in patients with PD‐L1 CPS ≥5. Hierarchically tested secondary endpoints included OS in patients with PD‐L1 CPS ≥1 and in all randomized patients. Other secondary endpoints that were not formally tested included PFS and ORR per BICR at different PD‐L1 CPS cutoffs and in all randomized patients. Prespecified exploratory endpoints included DOR per BICR, landmark survival rates, biomarkers that were potentially associated with efficacy and safety and tolerability. The Chinese subgroup analysis includes data from the primary, secondary and key exploratory endpoints, but the endpoint analyses are descriptive.

### 2.5 Assessments

Incidence of death, treatment‐related adverse events (TRAEs), serious adverse events, TRAEs leading to discontinuation and TRAEs with potential immunologic etiology were assessed using the Medical Dictionary for Regulatory Activities version 23.0, and adverse events (AEs) were graded in severity using the National Cancer Institute Common Terminology Criteria for Adverse Events version 4.0. AEs included events reported between the first dose and 30 days after the last dose of study therapy. Treatment‐relatedness could refer to nivolumab, at least one chemotherapy component or both in the nivolumab plus chemotherapy group. Tumors were assessed by computed tomography or magnetic resonance imaging at baseline, then every 6 weeks from first dose for 48 weeks, then every 12 weeks until disease progression.

Tumor cell PD‐L1 expression and PD‐L1 CPS were assessed using the Dako PD‐L1 IHC 28‐8 pharmDx assay (Dako, an Agilent Technologies Inc. company, Santa Clara, CA) at two central laboratories. Tumor cell PD‐L1 expression was defined as the percentage of viable tumor cells with partial or complete membrane staining in at least 100 viable tumor cells. CPS was generated by re‐scoring PD‐L1 stained slides and was defined as the number of PD‐L1‐positive tumor cells with partial or complete membrane staining, plus lymphocytes and macrophages with membrane staining, intracellular staining or both, divided by the total viable tumor cells multiplied by 100.

The all randomized patient population included all patients that were concurrently randomized to receive nivolumab plus chemotherapy or chemotherapy. The primary population included all randomized patients with PD‐L1 CPS ≥5. ORR was evaluated for all randomized patients with at least one measurable or target lesion at baseline. The safety population included all patients that received at least one dose of assigned study treatment.

### 2.6 Statistical analyses

Statistical analyses were performed using SAS version 9.4 (Cary, NC). For the Chinese subgroup patient population, a two‐sided stratified log‐rank test was not performed for OS and PFS as described for the global population.^18^ Only descriptive analyses were conducted for the Chinese subgroup patient population. HRs for OS and PFS were calculated using a stratified Cox proportional hazards model with treatment as the sole covariate. The patients from this subgroup were from Asia, so region was not used as a stratification factor in the analysis. The two‐sided 95% confidence intervals (CIs) for the HR were provided. Median OS, PFS and duration of response (DOR) for each treatment group were estimated using the Kaplan‐Meier product limit method. The two‐sided 95% CI for the median in each treatment group was calculated using the log‐log transformation method. PFS and OS rate estimates at fixed time points were derived from the Kaplan‐Meier estimate, with 95% CIs derived based on the Greenwood formula for variance derivation and on log‐log transformation applied to the survivor function. For the OS analyses in prespecified subgroups, the HR and corresponding 95% CI for nivolumab plus chemotherapy vs chemotherapy were calculated using an unstratified Cox proportional hazards model with treatment as the covariate.

## 3 RESULTS

### 3.1 Patients

**FIGURE 1:**
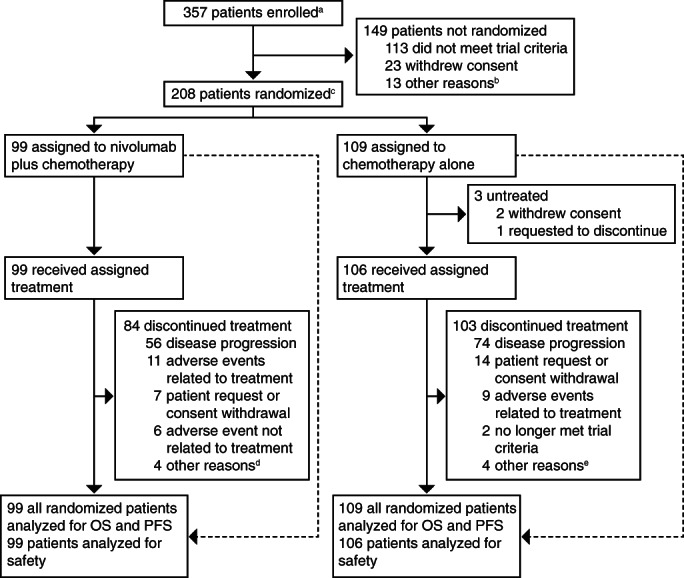
Trial profile for Chinese patients. OS, overall survival; PFS, progression‐free survival. ^a^The frequency of patients enrolled but not randomized does not reflect the actual screen failure rate as patients were randomized to three treatment groups, and this figure only displays randomization to nivolumab plus chemotherapy and chemotherapy groups. ^b^Poor/non‐compliance (n = 4), death (n = 2), adverse event (n = 1), administrative reason by sponsor (n = 1), other (n = 5). ^c^Patients concurrently randomized to the nivolumab plus chemotherapy or chemotherapy groups. ^d^Completed treatment as per protocol (n = 3), patient no longer met trial criteria (n = 1). ^e^Maximum clinical benefit (n = 1), other (n = 3)

From April 2017 to May 2019, 357 Chinese patients were assessed for eligibility in CheckMate 649. Among these, 208 patients were randomized to receive nivolumab plus chemotherapy (n = 99) or chemotherapy alone (n = 109); 99 patients in the nivolumab plus chemotherapy group and 106 patients in the chemotherapy group received at least one dose of the assigned treatment (Figure 1). 83% of patients received XELOX, and 17% of patients received FOLFOX across both treatment groups. Patients had either GC (89%) or GEJC (11%); no randomized patients had EAC. Of the 208 patients, 156 (75%) had PD‐L1 CPS ≥5 (nivolumab plus chemotherapy, 75/99 [76%]; chemotherapy, 81/109 [74%]) and 183 (88%) had PD‐L1 CPS ≥1 (nivolumab plus chemotherapy, 89/99 [90%]; chemotherapy, 94/109 [86%]). Baseline characteristics were balanced across treatment groups and consistent in both patients whose tumors expressed PD‐L1 CPS ≥5 and in the all randomized population (Table 1). Median follow‐up for OS (time from randomization to last known date alive or death) was 14.0 months for patients receiving nivolumab plus chemotherapy and 9.9 months for patients receiving chemotherapy alone. In the nivolumab plus chemotherapy group, 84 patients discontinued treatment. In the chemotherapy group, 103 patients discontinued treatment. In both treatment groups, disease progression was the most common reason for treatment discontinuation (nivolumab plus chemotherapy, n = 56 [57%]; chemotherapy, n = 74 [70%]; Figure 1).

**TABLE 1:** Baseline demographics and clinical characteristics

	Patients whose tumors express PD‐L1 CPS ≥5		All randomized patients	
	Nivolumab plus chemotherapy (n = 75)	Chemotherapy (n = 81)	Nivolumab plus chemotherapy (n = 99)	Chemotherapy (n = 109)
Median age (range), years	61 (23‐77)	60 (29‐85)	61 (23‐83)	60 (21‐85)
<65	48 (64)	60 (74)	65 (66)	79 (72)
≥65	27 (36)	21 (26)	34 (34)	30 (28)
Sex				
Male	48 (64)	57 (70)	64 (65)	75 (69)
Female	27 (36)	24 (30)	35 (35)	34 (31)
Median body weight (range), kg	60 (40‐83)	60 (43‐99)	60 (39‐90)	59 (37‐99)
ECOG PS^a^				
0	18 (24)	24 (30)	24 (24)	29 (27)
1	57 (76)	57 (70)	75 (76)	80 (73)
Primary tumor location at initial diagnosis^b^				
GC	67 (89)	70 (86)	90 (91)	96 (88)
GEJC	8 (11)	11 (14)	9 (9)	13 (12)
Tumor cell PD‐L1 expression				
<1%^c^	58 (77)	59 (73)	80 (81)	87 (80)
≥1%	17 (23)	22 (27)	19 (19)	22 (20)
Previous surgery				
Yes	14 (19)	16 (20)	23 (23)	20 (18)
No	61 (81)	65 (80)	76 (77)	89 (82)
Disease stage				
Metastatic	74 (99)	80 (99)	98 (99)	108 (99)
Locally advanced	1 (1)	1 (1)	1 (1)	1 (1)
Organs with metastases				
≤1	12 (16)	13 (16)	16 (16)	18 (17)
≥2	63 (84)	68 (84)	83 (84)	91 (83)
Site of metastases				
Liver	41 (55)	39 (48)	53 (54)	53 (49)
Peritoneum	11 (15)	10 (12)	21 (21)	17 (16)
Central nervous system	0	0	0	0
Signet ring cell carcinoma^d^				
Yes	6 (8)	5 (6)	10 (10)	8 (7)
No	69 (92)	76 (94)	89 (90)	101 (93)
Lauren classification				
Intestinal type	12 (16)	20 (25)	18 (18)	23 (21)
Diffuse type	14 (19)	14 (17)	17 (17)	18 (17)
Mixed	11 (15)	8 (10)	13 (13)	12 (11)
Unknown	38 (51)	39 (48)	51 (52)	56 (51)
*Helicobacter pylori*				
Yes	14 (19)	15 (19)	18 (18)	20 (18)
No	27 (36)	39 (48)	38 (38)	53 (49)
Unknown or not reported	34 (45)	27 (33)	43 (43)	36 (33)
Microsatellite instability status				
Microsatellite stable	74 (99)	79 (98)	97 (98)	107 (98)
Microsatellite instability‐high	0	2 (2)	1 (1)	2 (2)
Not reported or invalid	1 (1)	0	1 (1)	0
Chemotherapy regimen,^e^ n/N (%)				
FOLFOX	11/75 (15)	10/78 (13)	19/99 (19)	15/106 (14)
XELOX	64/75 (85)	68/78 (87)	80/99 (81)	91/106 (86)
PD‐L1 CPS				
<1	–	–	10 (10)	15 (14)
≥1	–	–	89 (90)	94 (86)
<5	–	–	24 (24)	28 (26)
≥5	–	–	75 (76)	81 (74)
Albumin				
<LLN	15 (20)	17 (21)	23 (23)	24 (22)
≥LLN	59 (79)	62 (77)	74 (75)	83 (76)
Not reported	1 (1)	2 (2)	2 (2)	2 (2)
Previous adjuvant or neoadjuvant therapy				
Adjuvant	6 (8)	4 (5)	10 (10)	6 (6)
Neoadjuvant	1 (1)	2 (2)	1 (1)	2 (2)
Metastatic disease	0	0	0	0

*Note*: Data are n (%) unless otherwise noted.

Abbreviations: CPS, combined positive score; ECOG PS, Eastern Cooperative Oncology Group performance status; FOLFOX, oxaliplatin, leucovorin and 5‐fluorouracil; GC, gastric cancer; GEJC, gastroesophageal junction cancer; LLN, lower limit of normal; PD‐L1, programmed death ligand 1; XELOX, capecitabine and oxaliplatin.

^a^ Based on case report form. All randomized patients had an ECOG PS of 0 or 1 based on interactive response technology.

^b^ No Chinese patients with esophageal adenocarcinoma were enrolled.

^c^ Includes indeterminate tumor cell PD‐L1 expression.

^d^ Per World Health Organization histological classification.

^e^ Patients who received at least one dose of the assigned treatment.

### 3.2 Efficacy

**FIGURE 2:**
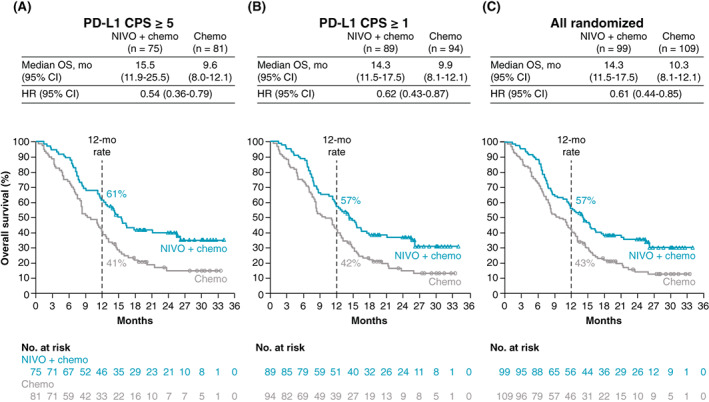
Overall survival in Chinese patients. Overall survival in patients with PD‐L1 CPS ≥5 (A), PD‐L1 CPS ≥1 (B) and in all randomized patients (C). Chemo, chemotherapy; CI, confidence interval; CPS, combined positive score; HR, hazard ratio; mo, months; NIVO, nivolumab; OS, overall survival; PD‐L1, programmed death ligand 1

After a minimum follow‐up (time from concurrent randomization of the last patient to data cutoff [May 27, 2020]) of 12.1 months, Chinese patients with PD‐L1 CPS ≥5 in the nivolumab plus chemotherapy group had a 46% reduction in risk of death and a 5.9‐month improvement in median OS (15.5 months [95% CI: 11.9‐25.5] vs 9.6 months [95% CI: 8.0‐12.1]; HR 0.54 [95% CI: 0.36‐0.79]) vs the chemotherapy group, respectively. Patients with PD‐L1 CPS ≥1 had a 38% reduction in risk of death (HR 0.62 [95% CI: 0.43‐0.87]) and a 4.4‐month improvement in median OS (14.3 months [95% CI: 11.5‐17.5] vs 9.9 months [95% CI: 8.1‐12.1]) vs the chemotherapy group. OS was also improved for all randomized patients in the nivolumab plus chemotherapy group, with a 4.0‐month improvement of median OS (14.3 months [95% CI: 11.5‐17.5] vs 10.3 months [95% CI: 8.1‐12.1]) as well as a 39% reduction in risk of death (HR 0.61 [95% CI: 0.44‐0.85]) vs the chemotherapy group. The 12‐month OS rates were numerically higher in the nivolumab plus chemotherapy group vs the chemotherapy group in all patient populations, at 61% (95% CI: 49‐71) vs 41% (95% CI: 30‐52) for PD‐L1 CPS ≥5, 57% (95% CI: 46‐67) vs 42% (95% CI: 32‐52) for PD‐L1 CPS ≥1 and 57% (95% CI: 46‐66) vs 43% (95% CI: 33‐52) for all randomized patients, respectively (Figure 2).

**FIGURE 3:**
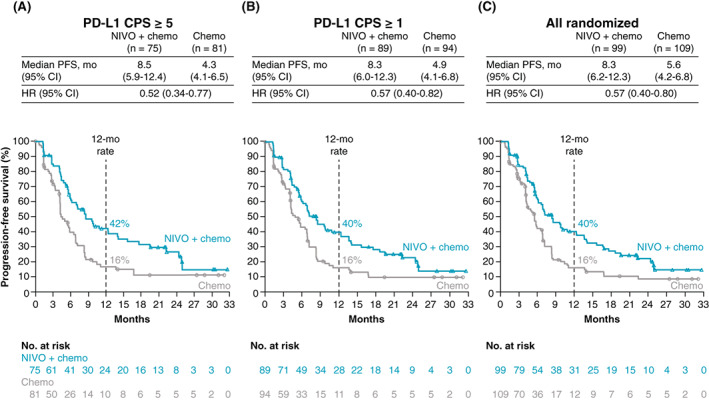
Progression‐free survival in Chinese patients. Progression‐free survival per BICR in patients with PD‐L1 CPS ≥5 (A) and PD‐L1 CPS ≥1 (B) and in all randomized patients (C). BICR, blinded independent central review; chemo, chemotherapy; CI, confidence interval; CPS, combined positive score; HR, hazard ratio; mo, months; NIVO, nivolumab; PD‐L1, programmed death ligand 1; PFS, progression‐free survival

The median PFS per BICR for patients with PD‐L1 CPS ≥5 treated with nivolumab plus chemotherapy vs chemotherapy was improved by 4.2 months (8.5 months [95% CI: 5.9‐12.4] vs 4.3 months [95% CI: 4.1‐6.5]; HR 0.52 [95% CI: 0.34‐0.77]) with a 48% reduction in risk of progression or death. The median PFS for patients with PD‐L1 CPS ≥1 was improved in the nivolumab plus chemotherapy group by 3.4 months (8.3 months [95% CI: 6.0‐12.3] vs 4.9 months [95% CI: 4.1‐6.8]; HR 0.57 [95% CI: 0.40‐0.82]) with a 43% reduction in risk of progression or death vs the chemotherapy group, respectively. Similarly, all randomized patients had a 2.7‐month improvement in median PFS with nivolumab plus chemotherapy (8.3 months [95% CI: 6.2‐12.3]) vs chemotherapy (5.6 months [95% CI: 4.2‐6.8]; HR 0.57 [95% CI: 0.40‐0.80]) with a 43% reduction in risk of progression or death. PFS rates at 12 months were numerically higher in the nivolumab plus chemotherapy group, at 42% (95% CI: 30‐53) vs 16% (95% CI: 9‐26) for PD‐L1 CPS ≥5, 40% (95% CI: 29‐50) vs 16% (95% CI: 9‐25) for PD‐L1 CPS ≥1 and 40% (95% CI: 30‐50) vs 16% (95% CI: 9‐25) for all randomized patients, respectively (Figure 3).

An objective response per BICR occurred in 47 patients (68% [95% CI: 56‐79]) treated with nivolumab plus chemotherapy and in 34 patients (48% [95% CI: 36‐60]) treated with chemotherapy in the PD‐L1 CPS ≥5 subgroup with 12 (17%) vs 9 patients (13%) experiencing a complete response, respectively. In all randomized patients, an objective response occurred in 58 patients (66% [95% CI: 55‐76]) treated with nivolumab plus chemotherapy and in 44 patients (45% [95% CI: 35‐56]) treated with chemotherapy, and complete responses occurred in 13 (15%) and 9 patients (9%), respectively (Supplementary Table S1). The median DOR was 11.0 months (95% CI: 7.2‐23.4) with nivolumab plus chemotherapy and 6.9 months (95% CI: 3.9‐8.5) with chemotherapy for patients with PD‐L1 CPS ≥5, and 12.2 months (95% CI: 7.2‐18.1) and 5.6 months (95% CI: 4.4‐8.3) for nivolumab plus chemotherapy vs chemotherapy in all randomized patients (Supplementary Figure S1).

The HRs for OS consistently favored the nivolumab plus chemotherapy group over the chemotherapy alone group across multiple prespecified subgroups (Supplementary Figure S2). Analysis of OS, PFS and ORR across PD‐L1 CPS subgroups also showed clinical benefit with nivolumab plus chemotherapy vs chemotherapy; enrichment was observed at higher cutoffs (Supplementary Figure S3).

Among all randomized patients, 47% in the nivolumab plus chemotherapy group and 59% in the chemotherapy group received subsequent therapy; 3% and 10% received subsequent immunotherapy, respectively (Supplementary Table S2).

### 3.3 Exposure and safety

Among all treated Chinese patients, the median (range) duration of treatment in the nivolumab plus chemotherapy group was 6.3 months (0.1‐32.6) for patients receiving nivolumab plus XELOX and 6.5 months (1.0‐30.0) for patients receiving nivolumab plus FOLFOX, while the median duration of treatment was 4.0 months (0.0‐29.2) and 3.9 months (0.8‐30.3) for patients receiving XELOX or FOLFOX alone, respectively. The duration of chemotherapy treatment was similar when comparing nivolumab plus chemotherapy to chemotherapy with the same backbone (Supplementary Table S3). The most common any‐grade TRAEs in both treatment groups were nausea, decreased platelet count, decreased white blood cell count, decreased neutrophil count, anemia, vomiting and increased aspartate aminotransferase. Any‐grade TRAEs occurred in 98 (99%) patients in the nivolumab plus chemotherapy group and in 100 (94%) patients in the chemotherapy group, and grade 3 or 4 TRAEs occurred in 64 (65%) and 53 (50%) patients in each group, respectively. Any‐grade serious TRAEs were observed in 26 (26%) patients (grade 3‐4: 19 [19%] patients) in the nivolumab plus chemotherapy group and 14 (13%) patients (grade 3‐4: 11 [10%] patients) in the chemotherapy group. Any‐grade TRAEs leading to discontinuation occurred in 50 (51%) patients in the nivolumab plus chemotherapy group and 27 (25%) patients in the chemotherapy group (Table 2). One death in the nivolumab plus chemotherapy group (infection per investigator) and one death (diarrhea per investigator) in the chemotherapy group was considered treatment related. Most TRAEs with potential immunologic etiology were grade 1‐2; grade 3‐4 events occurred in ≤5% of patients in the nivolumab plus chemotherapy group (Supplementary Table S4). Dose delays or reductions stemming from any‐grade TRAEs occurred in 74 (75%) patients in the nivolumab plus chemotherapy group and in 56 (53%) patients in the chemotherapy group.

**TABLE 2:** Summary of treatment‐related adverse events in all patients

	Nivolumab plus chemotherapy (n = 99)^a^		Chemotherapy (n = 106)^a^	
	Any grade	Grade 3–4	Any grade	Grade 3–4
All events	98 (99)	64 (65)	100 (94)	53 (50)
Serious events	26 (26)	19 (19)	14 (13)	11 (10)
Events leading to discontinuation	50 (51)	20 (20)	27 (25)	11 (10)
Any‐grade events in ≥10% of treated patients in either group				
Nausea	49 (49)	2 (2)	44 (42)	4 (4)
Platelet count decreased	48 (48)	7 (7)	40 (38)	12 (11)
White blood cell count decreased	46 (46)	6 (6)	37 (35)	2 (2)
Neutrophil count decreased	42 (42)	17 (17)	38 (36)	12 (11)
Anemia	39 (39)	6 (6)	38 (36)	3 (3)
Vomiting	38 (38)	1 (1)	33 (31)	5 (5)
Aspartate aminotransferase increased	35 (35)	1 (1)	33 (31)	1 (1)
Thrombocytopenia	31 (31)	7 (7)	22 (21)	4 (4)
Decreased appetite	27 (27)	2 (2)	25 (24)	4 (4)
Alanine aminotransferase increased	24 (24)	0	24 (23)	2 (2)
Leukopenia	23 (23)	1 (1)	21 (20)	4 (4)
Blood bilirubin increased	22 (22)	0	22 (21)	2 (2)
Neutropenia	21 (21)	6 (6)	19 (18)	8 (8)
Hypoesthesia	21 (21)	1 (1)	16 (15)	0
Pyrexia	20 (20)	0	7 (7)	0
Diarrhea	15 (15)	1 (1)	21 (20)	4 (4)
Hypoalbuminemia	15 (15)	0	10 (9)	0
Hemoglobin decreased	14 (14)	2 (2)	21 (20)	3 (3)
Palmar‐plantar erythrodysesthesia	12 (12)	2 (2)	15 (14)	1 (1)
Blood alkaline phosphatase increased	11 (11)	1 (1)	7 (7)	1 (1)
Hypothyroidism	11 (11)	0		
Fatigue	10 (10)	4 (4)	9 (8)	0
Lipase increased	10 (10)	3 (3)	6 (6)	2 (2)
Asthenia	10 (10)	1 (1)	6 (6)	0
Malaise	9 (9)	2 (2)	17 (16)	0
Weight decreased	9 (9)	1 (1)	12 (11)	0

*Note*: Data are n (%). One death in the nivolumab plus chemotherapy and one death in the chemotherapy group were considered treatment related. Treatment‐related deaths were reported regardless of timeframe.

^a^ Patients who received at least one dose of study drug. Includes events reported between first dose and 30 days after last dose of trial therapy. Treatment‐relatedness in the nivolumab plus chemotherapy group refers to nivolumab at least one chemotherapy component or both. Adverse events were assessed according to the National Cancer Institute Common Terminology Criteria for Adverse Events version 4.0 and Medical Dictionary for Regulatory Activities version 23.0. There were no grade 5 events in either treatment group.

## 4 DISCUSSION

Chinese patients who received nivolumab plus chemotherapy experienced clinically meaningful improvements in OS and PFS per BICR vs chemotherapy alone in the PD‐L1 CPS ≥5 and PD‐L1 CPS ≥1 groups and in the all randomized population. There was early and sustained separation of OS and PFS curves and a higher percentage of patients alive at 12 months in the nivolumab plus chemotherapy group vs the chemotherapy group, suggesting rapid and durable clinical benefit with nivolumab plus chemotherapy. OS consistently favored nivolumab plus chemotherapy vs chemotherapy across multiple prespecified subgroups, including by PD‐L1 CPS subgroups. Objective response rates were higher and responses were more durable in the nivolumab plus chemotherapy group compared with those in the chemotherapy group for patients with PD‐L1 CPS ≥5 and for all randomized patients. Nivolumab plus chemotherapy had an acceptable safety profile and was consistent with the known safety profiles of the individual treatment components with no new safety signals identified.^15^, ^18^, ^23^, ^24^, ^25^, ^26^

In the global study population (n = 1581), nivolumab plus chemotherapy demonstrated superior OS vs chemotherapy with a 29% reduction in the risk of death and 3.3‐month improvement in median OS for patients with PD‐L1 CPS ≥5, and a 20% reduction in the risk of death and 2.2‐month improvement in median OS for all randomized patients. Clinically meaningful OS benefit with nivolumab plus chemotherapy vs chemotherapy was also observed in the Chinese subgroup (n = 208) with a 46% reduction in risk of death and 5.9‐month improvement in median OS in patients with PD‐L1 CPS ≥5 and a 39% reduction in risk of death and 4.0‐month improvement in median OS in all randomized patients. While median OS and PFS in the chemotherapy group were shorter in Chinese patients compared with the global study population these were consistent with reports in other global trials^13^, ^14^, ^16^ and likely due to differences in baseline disease characteristics and a higher proportion of Chinese patients receiving subsequent therapies. The same trends in OS were observed in patients with PD‐L1 CPS ≥1. Other efficacy data including PFS, ORR and DOR (all per BICR) were also consistent with the global study population.

Safety was comparable between the Chinese subgroup and global study population with grade 3 to 4 TRAEs reported for 65% of Chinese patients and 59% of the global study population treated with nivolumab plus chemotherapy. However, rates of any‐grade TRAEs leading to discontinuation from nivolumab plus chemotherapy were higher in Chinese patients than the global study population (51% and 36% of patients, respectively). The type and severity of TRAEs observed in Chinese patients were similar to those observed in the global study population. Despite the increased incidence of TRAEs leading to discontinuation, median duration of treatment was comparable between the global study population and the Chinese subgroup, indicating that TRAEs leading to discontinuation likely did not have a substantial effect on treatment duration in this subgroup.^18^

While it is important to note that this analysis was not designed as a formal comparison with the global study population, differences in baseline disease characteristics and practice patterns, including subsequent therapies, were observed. Chinese patients, when compared with the global study population, respectively, had numerically higher rates of ECOG PS 1 (75% vs 58%) and GC as the primary tumor location at initial diagnosis (89% vs 70%). In the nivolumab plus chemotherapy group, a higher percentage of Chinese patients received XELOX compared with the global study population (81% vs 46%, respectively). The rate of subsequent therapy in the Chinese subgroup was also higher than the overall population in both the nivolumab plus chemotherapy (47% vs 38%, respectively) and chemotherapy (59% vs 41%) groups.^18^

The randomized phase III trial ORIENT‐16 evaluated the PD‐1 inhibitor sintilimab plus chemotherapy vs chemotherapy in Chinese patients with previously untreated, unresectable advanced, recurrent or metastatic GC/GEJC.^27^ Sintilimab plus chemotherapy demonstrated OS and PFS benefit in patients with PD‐L1 CPS ≥5 and all randomized patients compared with chemotherapy. The safety profile was manageable, and no new safety signals were identified. In the phase III ATTRACTION‐4 trial of nivolumab plus chemotherapy vs chemotherapy in patients from Japan, South Korea and Taiwan with advanced GC/GEJC, nivolumab plus chemotherapy resulted in significantly improved PFS but not OS.^28^ Nivolumab plus chemotherapy also demonstrated higher ORR, longer DOR and a manageable safety profile vs chemotherapy. In contrast, both OS and PFS benefits were observed with nivolumab plus chemotherapy vs chemotherapy in CheckMate 649.^28^ The disparate OS results between CheckMate 649 and ATTRACTION‐4 may be due to the higher proportion of patients from ATTRACTION‐4 receiving subsequent systemic therapies; in particular, more patients in the chemotherapy groups received subsequent immunotherapy in ATTRACTION‐4 compared with CheckMate 649 (27% vs 8%). Subsequent therapy is more commonly given in Asian countries although there are regional differences within Asia in the use of subsequent therapies that could have an impact on OS outcomes.^18^, ^28^ Rates of subsequent systemic therapy in CheckMate 649 were comparable between the overall population and the Chinese subgroup, and rates of first subsequent immunotherapy were similar.^18^ These data further support the efficacy and safety of first‐line treatment with a PD‐1 inhibitor plus chemotherapy for Asian patients with advanced or metastatic GC/GEJC.

It is important to consider the potential limitations of this study and the Chinese subgroup analysis. CheckMate 649 was an open‐label trial. A lack of blinding may result in differences in assessments such as causality of adverse events. The open‐label design was determined to be appropriate due to the use of multiple treatments and dosing regimens. It was not expected that the management of TRAEs (with standard treatment algorithms) or centrally assessed primary endpoints (eg, PFS) would be affected by bias. The Chinese subgroup was relatively small in comparison to the global population (n = 208 vs n = 1581, respectively).^18^ These endpoint analyses are descriptive and no formal statistical testing was conducted. However, the clinically meaningful improvements of OS, PFS, ORR and DOR indicate clinical benefit of nivolumab plus chemotherapy vs chemotherapy in the Chinese subgroup.

## 5 CONCLUSIONS

In this preplanned analysis of Chinese patients enrolled in CheckMate 649, nivolumab plus chemotherapy resulted in clinically meaningful improvements in OS, PFS, ORR and DOR compared with chemotherapy alone, and the safety profile was acceptable and showed no new safety signals, consistent with results from the global study population.^18^ On the basis of data from CheckMate 649, nivolumab plus chemotherapy is now approved as a first‐line treatment for unresectable advanced or metastatic GC/GEJC/EAC in the United States and China^29^ and represents a new standard first‐line treatment for Chinese patients with advanced GC/GEJC/EAC.

## AUTHOR CONTRIBUTIONS

The work reported in the paper has been performed by the authors unless clearly specified in the text. Kohei Shitara, Mingshun Li and Ming Lei contributed to the conception and design of the study. Tianshu Liu, Yuxian Bai, Xiaoyan Lin, Wei Li, Jufeng Wang, Xiaochun Zhang, Hongming Pan, Chunmei Bai, Li Bai, Ying Cheng, Jingdong Zhang, Haijun Zhong, Yi Ba, Wenwei Hu, Ruihua Xu, Weijian Guo, Shukui Qin, Nong Yang, Jianwei Lu and Lin Shen recruited and/or treated patients and gathered clinical data on efficacy and safety. Mingshun Li analyzed the clinical data, Ming Lei performed biomarker analyses and Tian Chen conducted statistical analyses. Ming Lei, Mingshun Li, Nicole Bao and Tian Chen verified the data. All authors interpreted the data. All authors had access to all the data in the study, participated in developing or reviewing the manuscript and provided final approval to submit the manuscript for publication. The work reported in the paper has been performed by the authors unless clearly specified in the text.

## CONFLICT OF INTEREST

Kohei Shitara reports grants and personal fees from Astellas Pharma, Eli Lilly and Company, Ono Pharmaceutical, Daiichi Sankyo, Taiho Pharmaceutical and Merck Pharmaceutical; grants from Dainippon Sumitomo Pharma, Chugai Pharma, Medi Science and Eisai; and personal fees from Bristol Myers Squibb, Takeda Pharmaceuticals, Pfizer, Novartis, AbbVie, Yakult, GlaxoSmithKline, Amgen and Boehringer Ingelheim. Ming Lei reports employment with Bristol Myers Squibb and ownership of stock in Bristol Myers Squibb. Mingshun Li reports employment with Bristol Myers Squibb. Tian Chen reports holding a patent to combination therapy with anti‐IL‐8 antibodies and anti‐PD‐1 antibodies for treating cancer. Lin Shen reports grants from Beijing Xiantong Biomedical Technology Co. Ltd, Qilu Pharmaceutical Co. Ltd, Zaiding Pharmaceutical (Shanghai) Co. Ltd, Jacobio Pharmaceuticals Co. Ltd, Beihai Kangcheng (Beijing) Medical Technology Co. Ltd and Boehringer Ingelheim; and consulting fees from Harbor BioMed and Merck. All other authors declare no competing interests.

## ETHICS STATEMENT

This study was conducted in accordance with the trial protocol and with Good Clinical Practice guidelines developed by the International Council for Harmonisation. Written informed consent was obtained from all patients per the Declaration of Helsinki principles. The study is registered at ClinicalTrials.gov NCT02872116.

## Supporting information


**APPENDIX S1** Supporting Information

## Data Availability

Bristol Myers Squibb will honor legitimate requests for clinical trial data from qualified researchers. Data will be shared with external researchers whose proposed use of the data has been approved. Complete de‐identified patient data sets will be eligible for sharing 2 years after completion of the CheckMate 649 study. Before data are released the researcher(s) must sign a Data Sharing Agreement after which the de‐identified and anonymized datasets can be accessed within a secured portal. Bristol Myers Squibb policy on data sharing may be found at https://www.bms.com/researchers-and-partners/independent-research/data-sharing-request-process.html. Further information is available from the corresponding author upon request.

## References

[1] Sung H , Ferlay J , Siegel RL , et al. Global cancer statistics 2020: GLOBOCAN estimates of incidence and mortality worldwide for 36 cancers in 185 countries. CA Cancer J Clin. 2021;71(3):209‐249. 33538338 10.3322/caac.21660

[2] International Agency for Research on Cancer 2020. China cancer incidence and mortality 2020: GLOBOCAN fact sheet 2020.

[3] Ajani JA , Lee J , Sano T , Janjigian YY , Fan D , Song S . Gastric adenocarcinoma. Nat Rev Dis Primers. 2017;3(1):17036. 28569272 10.1038/nrdp.2017.36

[4] Arnold M, Ferlay J , van Berge Henegouwen MI , Soerjomataram I . Global burden of oesophageal and gastric cancer by histology and subsite in 2018. Gut. 2020;69(9):1564. 32606208 10.1136/gutjnl-2020-321600

[5] National Comprehensive Cancer Network . Gastric cancer (version 3.2021). https://www.nccn.org/professionals/physician_gls/pdf/gastric.pdf

[6] National Comprehensive Cancer Network . Esophageal and esophagogastric junction cancers (version 3.2021). https://www.nccn.org/professionals/physician_gls/pdf/gastric.pdf 10.6004/jnccn.2015.0028 25691612

[7] Wang FH , Shen L , Li J , et al. The Chinese Society of Clinical Oncology (CSCO): clinical guidelines for the diagnosis and treatment of gastric cancer. Cancer Commun (Lond). 2019;39(1):10. 30885279 10.1186/s40880-019-0349-9 PMC6423835

[8] National Health Commission of the People's Republic of China . Chinese guidelines for diagnosis and treatment of esophageal carcinoma 2018 (English version). Chin J Cancer Res. 2019;31(2):223‐258. 31156297 10.21147/j.issn.1000-9604.2019.02.01 PMC6513746

[9] Smyth EC , Verheij M , Allum W , Cunningham D , Cervantes A , Arnold D . Gastric cancer: ESMO clinical practice guidelines for diagnosis, treatment and follow‐up. Ann Oncol. 2016;27(suppl 5):v38‐v49. 27664260 10.1093/annonc/mdw350

[10] Japanese Gastric Cancer Association . Japanese gastric cancer treatment guidelines 2018 (5th edition). Gastric Cancer. 2021;24(1):1‐21. 32060757 10.1007/s10120-020-01042-y PMC7790804

[11] Salem ME , Puccini A , Xiu J , et al. Comparative molecular analyses of esophageal squamous cell carcinoma, esophageal adenocarcinoma, and gastric adenocarcinoma. Oncologist. 2018;23(11):1319‐1327. 29866946 10.1634/theoncologist.2018-0143 PMC6291329

[12] Cancer Genome Atlas Research Network . Integrated genomic characterization of oesophageal carcinoma. Nature. 2017;541(7636):169‐175. 28052061 10.1038/nature20805 PMC5651175

[13] Lordick F , Kang YK , Chung HC , et al. Capecitabine and cisplatin with or without cetuximab for patients with previously untreated advanced gastric cancer (EXPAND): a randomised, open‐label phase 3 trial. Lancet Oncol. 2013;14(6):490‐499. 23594786 10.1016/S1470-2045(13)70102-5

[14] Catenacci DVT , Tebbutt NC , Davidenko I , et al. Rilotumumab plus epirubicin, cisplatin, and capecitabine as first‐line therapy in advanced MET‐positive gastric or gastro‐oesophageal junction cancer (RILOMET‐1): a randomised, double‐blind, placebo‐controlled, phase 3 trial. Lancet Oncol. 2017;18(11):1467‐1482. 28958504 10.1016/S1470-2045(17)30566-1 PMC5898242

[15] Shah MA , Bang YJ , Lordick F , et al. Effect of fluorouracil, leucovorin, and oxaliplatin with or without onartuzumab in HER2‐negative, MET‐positive gastroesophageal adenocarcinoma: the METGastric randomized clinical trial. JAMA Oncol. 2017;3(5):620‐627. 27918764 10.1001/jamaoncol.2016.5580 PMC5824210

[16] Fuchs CS , Shitara K , Di Bartolomeo M , et al. Ramucirumab with cisplatin and fluoropyrimidine as first‐line therapy in patients with metastatic gastric or junctional adenocarcinoma (RAINFALL): a double‐blind, randomised, placebo‐controlled, phase 3 trial. Lancet Oncol. 2019;20(3):420‐435. 30718072 10.1016/S1470-2045(18)30791-5

[17] The Chinese Society of Clinical Oncology (CSCO) . The Chinese Society of Clinical Oncology (CSCO) Guideline for Gastric Cancer. Beijing: People's Medical Publishing House; 2021.

[18] Janjigian YY , Shitara K , Moehler M , et al. First‐line nivolumab plus chemotherapy versus chemotherapy alone for advanced gastric, gastro‐oesophageal junction, and oesophageal adenocarcinoma (CheckMate 649): a randomised, open‐label, phase 3 trial. Lancet. 2021;398(10294):27‐40. 34102137 10.1016/S0140-6736(21)00797-2 PMC8436782

[19] Peng L, Qin B‐D , Xiao K , et al. A meta‐analysis comparing responses of Asian versus non‐Asian cancer patients to PD‐1 and PD‐L1 inhibitor‐based therapy. Oncoimmunology 2020;9(1):1781333. 32923143 10.1080/2162402X.2020.1781333 PMC7458616

[20] Chen C , Zhang F , Zhou N , et al. Efficacy and safety of immune checkpoint inhibitors in advanced gastric or gastroesophageal junction cancer: a systematic review and meta‐analysis. Oncoimmunology 2019;8(5):e1581547. 31069144 10.1080/2162402X.2019.1581547 PMC6492970

[21] Lin SJ , Gagnon‐Bartsch JA , Tan IB , et al. Signatures of tumour immunity distinguish Asian and non‐Asian gastric adenocarcinomas. Gut. 2015;64(11):1721‐1731. 25385008 10.1136/gutjnl-2014-308252 PMC4680172

[22] Strong VE, Wu A‐W , Selby LV , et al. Differences in gastric cancer survival between the U.S. and China. J Surg Oncol. 2015;112(1):31‐37. 26175203 10.1002/jso.23940 PMC4667726

[23] Kang YK , Boku N , Satoh T , et al. Nivolumab in patients with advanced gastric or gastro‐oesophageal junction cancer refractory to, or intolerant of, at least two previous chemotherapy regimens (ONO‐4538‐12, ATTRACTION‐2): a randomised, double‐blind, placebo‐controlled, phase 3 trial. Lancet. 2017;390(10111):2461‐2471. 28993052 10.1016/S0140-6736(17)31827-5

[24] Boku N , Ryu MH , Oh DY , et al. LBA7_PR Nivolumab plus chemotherapy versus chemotherapy alone in patients with previously untreated advanced or recurrent gastric/gastroesophageal junction (G/GEJ) cancer: ATTRACTION‐4 (ONO‐4538‐37) study. Ann Oncol. 2020;31:S1192.

[25] Al‐Batran SE , Hartmann JT , Probst S , et al. Phase III trial in metastatic gastroesophageal adenocarcinoma with fluorouracil, leucovorin plus either oxaliplatin or cisplatin: a study of the Arbeitsgemeinschaft Internistische Onkologie. J Clin Oncol. 2008;26(9):1435‐1442. 18349393 10.1200/JCO.2007.13.9378

[26] Cunningham D , Starling N , Rao S , et al. Capecitabine and oxaliplatin for advanced esophagogastric cancer. N Engl J Med. 2008;358(1):36‐46. 18172173 10.1056/NEJMoa073149

[27] Xu J , Jiang H , Pan Y , et al. LBA53: Sintilimab plus chemotherapy (chemo) versus chemo as first‐line treatment for advanced gastric or gastroesophageal junction (G/GEJ) adenocarcinoma (ORIENT‐16): first results of a randomized, double‐blind, phase III study. Ann Oncol. 2021;32(5):S1283‐S1346.

[28] Kang Y‐K, Chen L‐T , Ryu M‐H , et al. Nivolumab plus chemotherapy versus placebo plus chemotherapy in patients with HER2‐negative, untreated, unresectable advanced or recurrent gastric or gastro‐oesophageal junction cancer (ATTRACTION‐4): a randomised, multicentre, double‐blind, placebo‐controlled, phase 3 trial. Lancet Oncol. 2022;23(2):234‐247. 35030335 10.1016/S1470-2045(21)00692-6

[29] OPDIVO (nivolumab) . US FDA prescribing information. Princeton, NJ: Bristol Myers Squibb; 2021.

