# Supplementary material for: First‐line nivolumab plus chemotherapy vs chemotherapy in patients with advanced gastric, gastroesophageal junction and esophageal adenocarcinoma: CheckMate 649 Chinese subgroup analysis
Source: Int J Cancer. 2022 Oct 31;152(4):749–60. doi: 10.1002/ijc.34296 (PMC10092493; doi:10.1002/ijc.34296)
Supplement: Supplementary file 1 — APPENDIX S1 Supporting Information [file IJC-152-749-s001.pdf]

Supplementary Material for

First-line nivolumab plus chemotherapy vs chemotherapy in patients with advanced gastric, gastroesophageal junction, and esophageal adenocarcinoma: CheckMate 649 Chinese subgroup analysis.

Tianshu Liu, Yuxian Bai, Xiaoyan Lin, Wei Li, Jufeng Wang, Xiaochun Zhang, Hongming Pan, Chunmei Bai, Li Bai, Ying Cheng, Jingdong Zhang, Haijun Zhong, Yi Ba, Wenwei Hu, Ruihua Xu, Weijian Guo, Shukui Qin, Nong Yang, Jianwei Lu, Kohei Shitara, Ming Lei, Mingshun Li, Nicole Bao, Tian Chen, Lin Shen

Table of Contents

Table S1 Objective and best overall response per BICR in Chinese patients 2
Table S2 Subsequent therapies in Chinese patients 3
Table S3 Exposure and disposition in Chinese patients 5
Table S4 Treatment-related adverse events with potential immunologic etiology in Chinese patients 6

Fig. S1 Duration of response in Chinese patients 7
Fig. S2 Prespecified subgroup analysis of OS in Chinese patients 8
Fig. S3 Subgroup analysis by PD-L1 CPS in Chinese patients 11

Supplemental Tables

TABLE S1 Objective and best overall response per BICR in Chinese patients

	**Patients whose tumors express PD-L1 CPS ≥ 5**		**All randomized patients**	
	**Nivolumab plus chemotherapy (n = 69)[a]**	**Chemotherapy (n = 71)[a]**	**Nivolumab plus chemotherapy (n = 88)[a]**	**Chemotherapy (n = 97)[a]**
ORR, n (%)	47 (68)	34 (48)	58 (66)	44 (45)
95% CI	(56-79)	(36-60)	(55-76)	(35-56)
Best overall response, n (%)				
Complete response	12 (17)	9 (13)	13 (15)	9 (9)
Partial response	35 (51)	25 (35)	45 (51)	35 (36)
Stable disease	16 (23)	18 (25)	22 (25)	29 (30)
Progressive disease	6 (9)	12 (17)	8 (9)	14 (14)
Not evaluable	0	7 (10)	0	10 (10)
Median time to response,[b] months (range)	1.5 (1.2-7.2)	1.4 (1.3-3.0)	1.5 (1.2-7.2)	1.4 (1.3-6.9)
Patients with ongoing response, n (%)[b]	13 (28)	6 (18)	15 (26)	6 (14)

Abbreviations: CPS, combined positive score; PD-L1, programmed death ligand 1.

[a]Patients with target lesion measurements at baseline per BICR assessment.

[b]Evaluated in patients who had an objective response.

TABLE S2 Subsequent therapies in Chinese patients

	**Patients whose tumors express PD-L1 CPS ≥ 5**		**All randomized patients**	
	**Nivolumab plus chemotherapy (n = 75)**	**Chemotherapy (n = 81)**	**Nivolumab plus chemotherapy (n = 99)**	**Chemotherapy (n = 109)**
Any subsequent therapy[a]	36 (48)	47 (58)	47 (47)	64 (59)
Radiotherapy	3 (4)	0	3 (3)	0
Surgery	1 (1)	1 (1)	2 (2)	2 (2)
Systemic anticancer therapy	35 (47)	47 (58)	45 (45)	64 (59)
Most frequent systemic anticancer therapies				
Taxanes				
Paclitaxel	17 (23)	30 (37)	22 (22)	41 (38)
Docetaxel	3 (4)	7 (9)	4 (4)	9 (8)
Platinum-based chemotherapy				
Oxaliplatin	5 (7)	9 (11)	6 (6)	10 (9)
Cisplatin	3 (4)	6 (7)	4 (4)	6 (6)
Fluoropyrimidine-based chemotherapy				
Gimeracil/ oteracil/tegafur	7 (9)	11 (14)	9 (9)	16 (15)
Capecitabine	6 (8)	2 (2)	8 (8)	2 (2)
Fluorouracil	1 (1)	4 (5)	1 (1)	5 (5)
Other chemotherapy				
Irinotecan	5 (7)	11 (14)	6 (6)	13 (12)
Raltitrexed	3 (4)	5 (6)	4 (4)	7 (6)
Targeted therapy				
Apatinib	10 (13)	15 (19)	12 (12)	18 (17)
Trastuzumab	2 (3)	2 (2)	2 (2)	2 (2)
Immunotherapy				
Nivolumab	1 (1)	2 (2)	1 (1)	3 (3)
	0	3 (4)	0	4 (4)
Pembrolizumab				
Toripalimab	1 (1)	2 (2)	1 (1)	3 (3)
Other	0	1 (1)	1 (1)	1 (1)

Note: Data are n (%).

Abbreviations: CPS, combined positive score; PD-L1, programmed death ligand 1.

[a]Patients could have received more than one type of therapy.

TABLE S3 Exposure and disposition in Chinese patients

	Median duration of treatment, months	Dose reduction or omission
Nivolumab plus chemotherapy (n = 99)		
Nivolumab plus XELOX (n = 80)	6.3 (0.1-32.6)	
Nivolumab[a] (mg)	5.8 (0.0-24.0)	
Oxaliplatin[b] (mg/m^2)	4.1 (0.0-9.7)	46 (58)
Capecitabine[c] (mg/m^2)	5.7 (0.1-32.6)	14 (18)
Nivolumab plus FOLFOX (n = 19)	6.5 (1.0-30.0)	
Nivolumab[a] (mg)	6.3 (1.0-23.9)	
Oxaliplatin[b] (mg/m^2)	4.8 (1.0-6.2)	11 (58)
Leucovorin (mg/m^2)	5.1 (1.0-29.9)	11 (58)
5-fluorouracil bolus[b] (mg/m^2)	5.2 (1.0-29.9)	11 (58)
5-fluorouracil continuous[b] (mg/m^2)	5.3 (1.0-30.0)	9 (47)
Chemotherapy (n = 106)		
XELOX (n = 91)	4.0 (0.0-29.2)	
Oxaliplatin[b] (mg/m^2)	3.5 (0.0-13.3)	29 (32)
Capecitabine[c] (mg/m^2)	3.9 (0.0-29.2)	16 (18)
FOLFOX (n = 15)	3.9 (0.8-30.3)	
Oxaliplatin[b] (mg/m^2)	3.9 (0.7-10.8)	8 (53)
Leucovorin (mg/m^2)	3.9 (0.7-30.2)	6 (40)
5-fluorouracil bolus[b] ($mg/m^{2)}$	3.9 (0.7-30.2)	6 (40)
5-fluorouracil continuous[b] (mg/m^2)	3.9 (0.8-30.3)	7 (47)

Note: Data are median (range).

Abbreviations: FOLFOX, oxaliplatin, leucovorin, and 5-fluorouracil; XELOX, capecitabine and oxaliplatin.

[a]Dose reductions were not allowed for nivolumab.

[b]Includes patients with at least one dose reduction.

[c]Includes patients with at least one omitted dose.

TABLE S4 Treatment-related adverse events with potential immunologic etiology in Chinese patients

TRAEs with potential immunologic etiology,[a] n (%)	Nivolumab plus chemotherapy (n = 99)		Chemotherapy (n = 106)	
	**Any grade**	**Grade 3-4**	**Any grade**	**Grade 3-4**
Endocrine	21 (21)	0	0	0
Gastrointestinal	15 (15)	1 (1)	21 (20)	4 (4)
Hepatic	57 (58)	4 (4)	53 (50)	6 (6)
Pulmonary	4 (4)	0	0	0
Renal	5 (5)	0	0	0
Skin	22 (22)	5 (5)	16 (15)	1 (<1)

[a]TRAEs with potential immunologic etiology includes events reported between first dose and 30 days after last dose of trial therapy according to National Cancer Institute Common Terminology Criteria for Adverse Events, version 4.0

Supplemental Figures

FIGURE S1 Duration of response in Chinese patients

(A) Patients with PD-L1 CPS ≥ 5

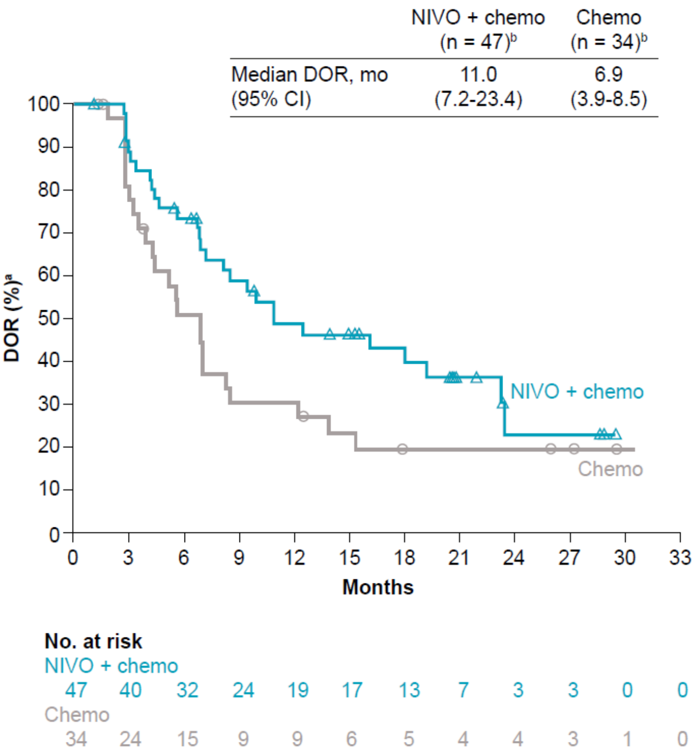

(B) All randomized patients

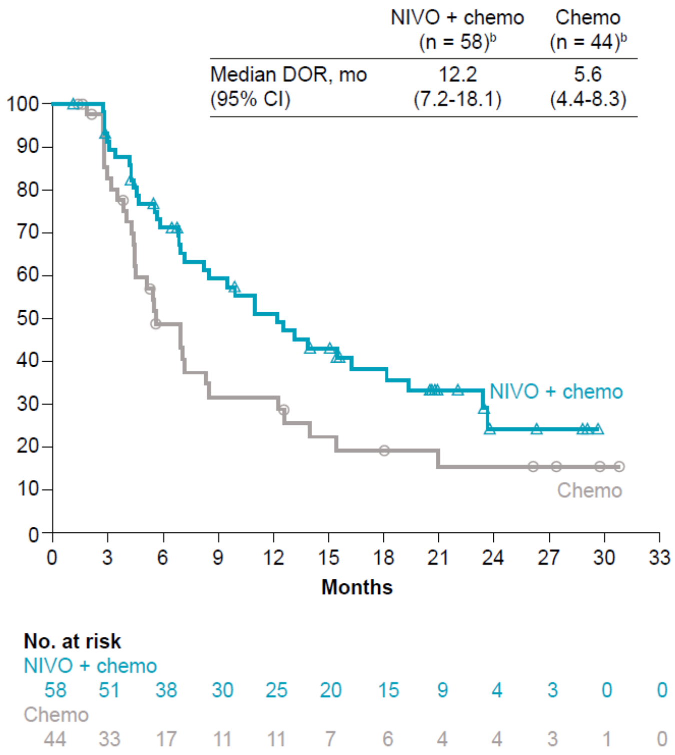

Abbreviations: CI, confidence interval; DOR, duration of response.

[a]Randomized patients who had target lesion measurements at baseline per BICR.

[b]Number of responders

FIGURE S2 Prespecified subgroup analysis of OS in Chinese patients

(A) Patients with PD-L1 CPS ≥ 5

Category (PD-L1 CPS ≥ 5)	Median, months		Unstratified HR (95% CI)
	NIVO + chemo	Chemo	
**Overall (N = 156)**	15.5	9.6	0.54 (0.37-0.79)
**Age, years**			
< 65 (n = 108)	13.9	9.9	0.64 (0.41-1.00)
≥ 65 (n = 48)	NR	8.3	0.43 (0.21-0.90)
**Sex**			
Male (n = 105)	15.6	11.1	0.52 (0.33-0.83)
Female (n = 51)	14.8	9.2	0.58 (0.30-1.11)
**ECOG PS**			
0 (n = 42)	17.1	12.6	0.84 (0.38-1.83)
1 (n = 114)	14.3	8.3	0.43 (0.28-0.67)
**Primary tumor location**			
GC (n = 137)	15.6	8.4	0.48 (0.32-0.72)
GEJC (n = 19)	10.5	17.9	NA[b]
**Liver metastases[a]**			
Yes (n = 80)	14.3	7.5	0.41 (0.24-0.68)
No (n = 75)	15.6	12.7	0.68 (0.39-1.20)
**Tumor cell PD-L1 expression**			
< 1% (n = 117)	14.3	11.3	0.64 (0.41-0.98)
≥ 1% (n = 39)	17.8	6.8	0.32 (0.14-0.73)
***Helicobacter pylori*[a]**			
Yes (n = 29)	NR	14.4	0.50 (0.19-1.34)
No (n = 66)	13.8	8.4	0.69 (0.40-1.19)
Unknown (n = 60)	16.4	8.3	0.44 (0.24-0.82)
**MSI status[a]**			
MSI-H (n = 2)	NA	8.8	NA[b]
MSS (n = 153)	14.8	10.5	0.56 (0.38-0.81)
**Chemotherapy regimen[c]**			
FOLFOX (n = 21)	11.7	10.3	0.74 (0.28-1.93)
XELOX (n = 132)	15.6	9.6	0.50 (0.33-0.76)

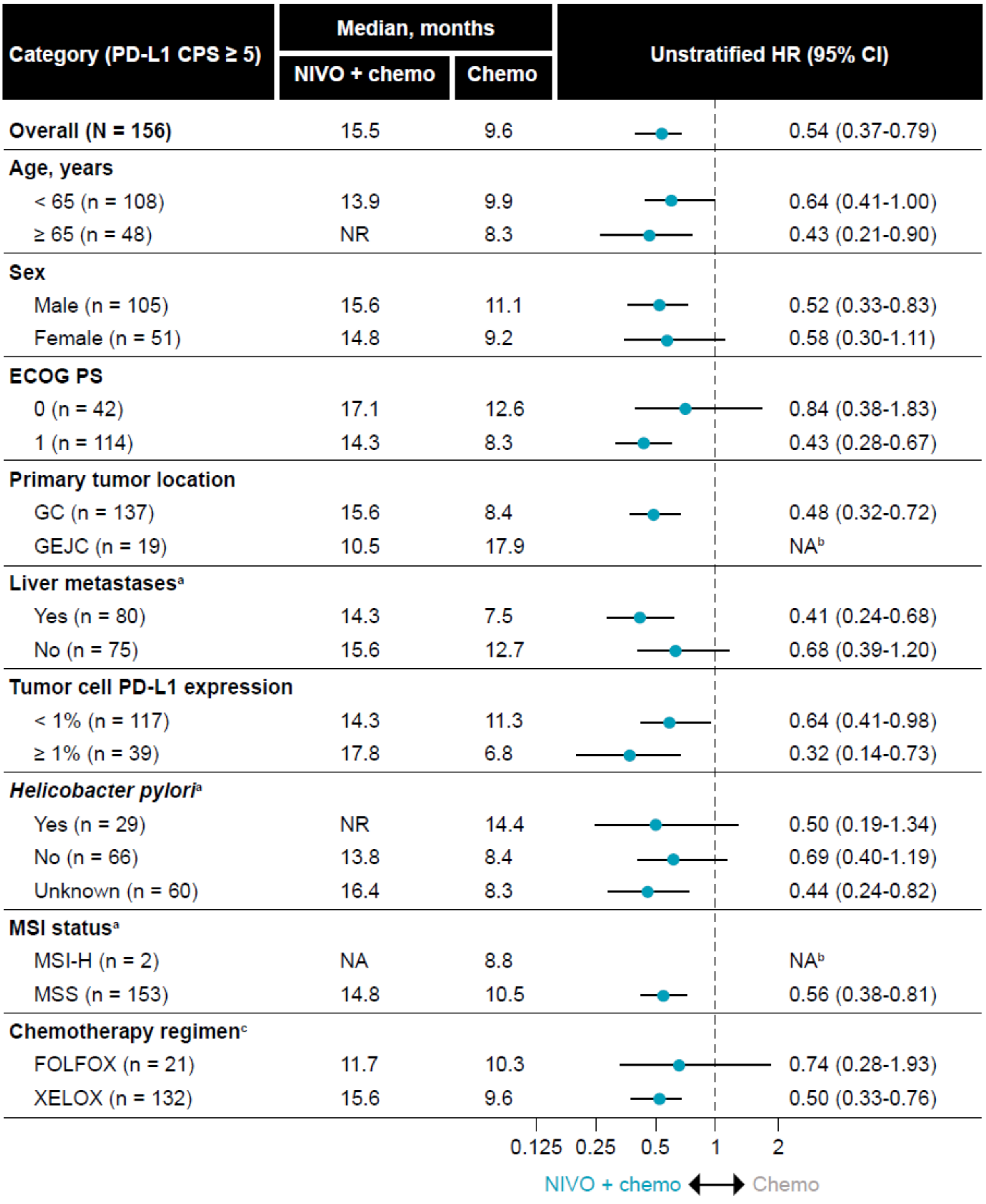

(B) All randomized patients

Category (all randomized)	Median, months		Unstratified HR (95% CI)
	NIVO + chemo	Chemo	
**Overall (N = 208)**	14.3	10.3	0.60 (0.43-0.82)
**Age, years**			
< 65 (n = 144)	11.9	10.5	0.68 (0.46-0.99)
≥ 65 (n = 64)	15.3	9.5	0.48 (0.26-0.86)
**Sex**			
Male (n = 139)	14.3	11.1	0.58 (0.39-0.86)
Female (n = 69)	11.9	9.2	0.62 (0.36-1.07)
**ECOG PS**			
0 (n = 53)	15.6	12.1	0.84 (0.43-1.66)
1 (n = 155)	14.0	8.3	0.52 (0.36-0.75)
**Primary tumor location**			
GC (n = 186)	14.3	8.5	0.55 (0.39-0.77)
GEJC (n = 22)	8.6	16.0	NA[b]
**Liver metastases[a]**			
Yes (n = 106)	14.2	8.1	0.48 (0.31-0.74)
No (n = 101)	14.8	12.4	0.75 (0.47-1.19)
**Tumor cell PD-L1 expression**			
< 1% (n = 167)	14.0	11.3	0.66 (0.46-0.94)
≥ 1% (n = 41)	15.6	6.8	0.39 (0.18-0.82)
***Helicobacter pylori*[a]**			
Yes (n = 38)	NR	12.1	0.51 (0.22-1.14)
No (n = 91)	12.5	9.5	0.76 (0.47-1.21)
Unknown (n = 78)	15.6	8.8	0.52 (0.30-0.89)
**MSI status[a]**			
MSI-H (n = 3)	NA	8.8	NA[b]
MSS (n = 204)	14.2	10.8	0.62 (0.45-0.86)
**Chemotherapy regimen[c]**			
FOLFOX (n = 34)	11.7	9.0	0.71 (0.33-1.53)
XELOX (n = 171)	15.3	10.6	0.56 (0.40-0.80)

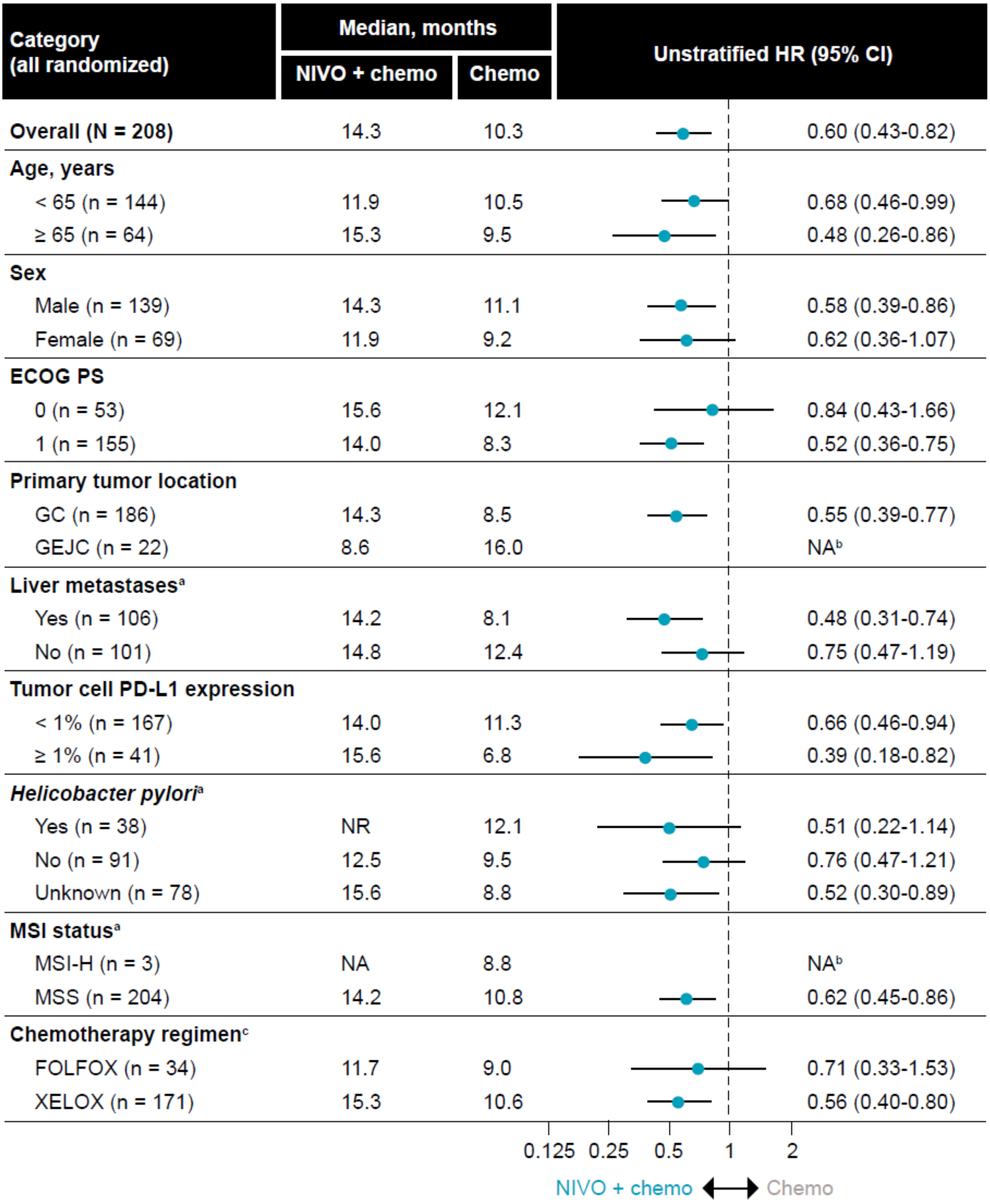

Note: HRs and corresponding 95% CIs for nivolumab plus chemotherapy relative to chemotherapy alone were calculated using a Cox model.

Abbreviations: CI, confidence interval; CPS, combined positive score; ECOG PS, Eastern Cooperative Oncology Group performance status; FOLFOX, oxaliplatin, leucovorin, and 5-fluorouracil; HR, hazard ratio; MSI, microsatellite instability; OS, overall survival; PD-L1, programmed death ligand 1; XELOX, capecitabine and oxaliplatin.

[a]Not reported for one patient.

[b]HR was not computed for subsets with fewer than 10 patients per treatment group.

[c]Patients who received at least one dose of the assigned treatment.

FIGURE S3 Subgroup analysis by PD-L1 CPS in Chinese patients

Survival

PD-L1 CPS[a]	Median, months		Unstratified HR[b] (95% CI)
	NIVO + chemo	Chemo	
**Overall survival**			
**Overall (N = 208)**	14.3	10.3	0.61 (0.44-0.85)
< 1 (n = 25)	11.8	10.6	0.69 (0.27-1.75)
≥ 1 (n = 183)	14.3	9.9	0.59 (0.42-0.82)
< 5 (n = 52)	10.1	11.0	0.81 (0.44-1.48)
≥ 5 (n = 156)	15.5	9.6	0.54 (0.37-0.79)
**Progression-free survival**			
**Overall (N = 208)**	8.3	5.6	0.57 (0.40-0.80)
< 1 (n = 25)	10.0	5.8	0.51 (0.18-1.45)
≥ 1 (n = 183)	8.3	4.9	0.59 (0.42-0.83)
< 5 (n = 52)	6.9	6.8	0.70 (0.36-1.34)
≥ 5 (n = 156)	8.5	4.3	0.55 (0.38-0.81)

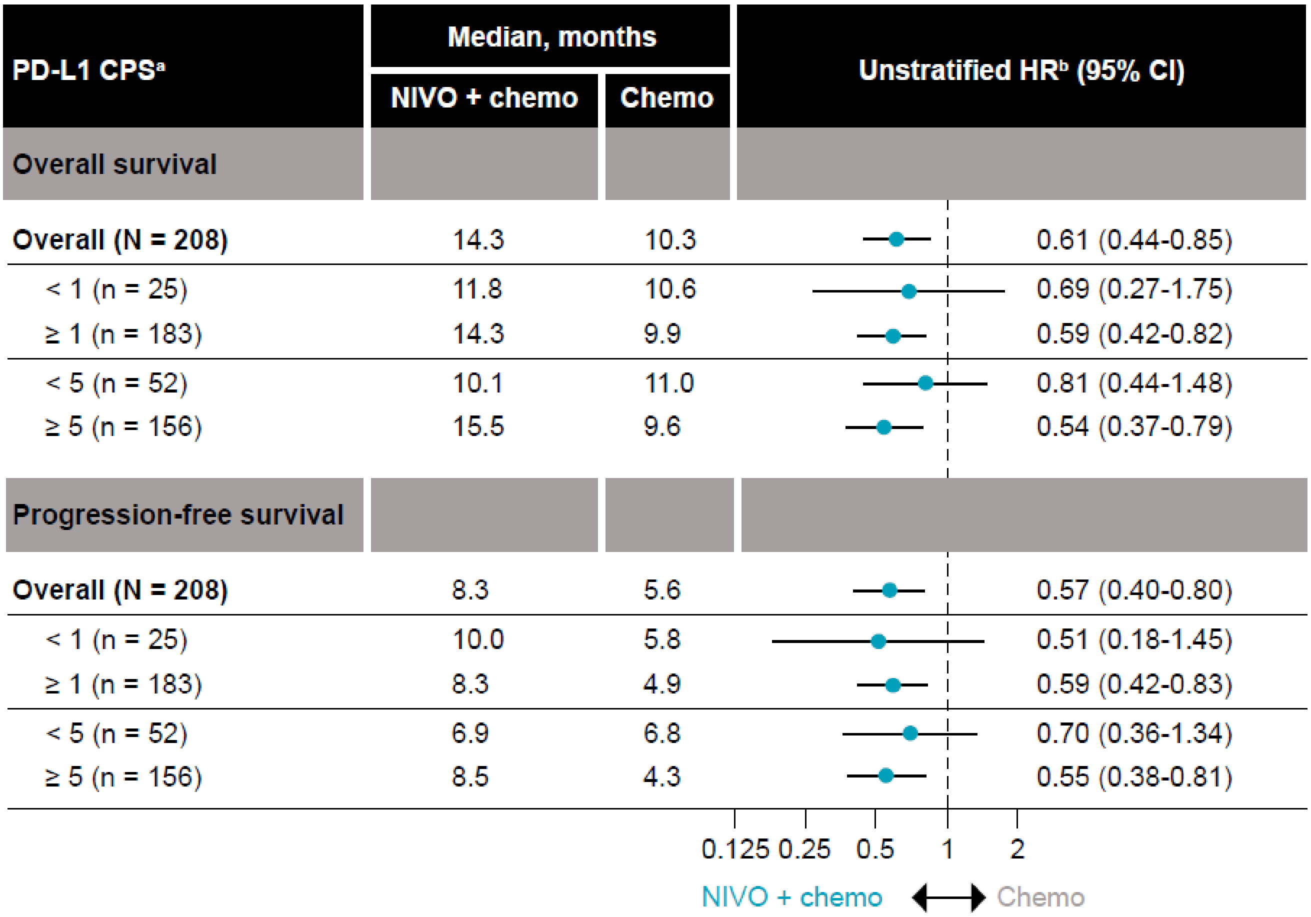

Objective response rate

PD-L1 CPS[c]	ORR, %		Unweighted ORR difference,[d] % (95% CI)
	NIVO + chemo	Chemo	
**Overall (N = 185)**	66	45	21 (7-35)
< 1 (n = 22)	75	36	39 (–3 to 66)
≥ 1 (n = 163)	65	47	18 (3-32)
< 5 (n = 45)	58	38	19 (–9 to 44)
≥ 5 (n = 140)	68	48	20 (4-35)

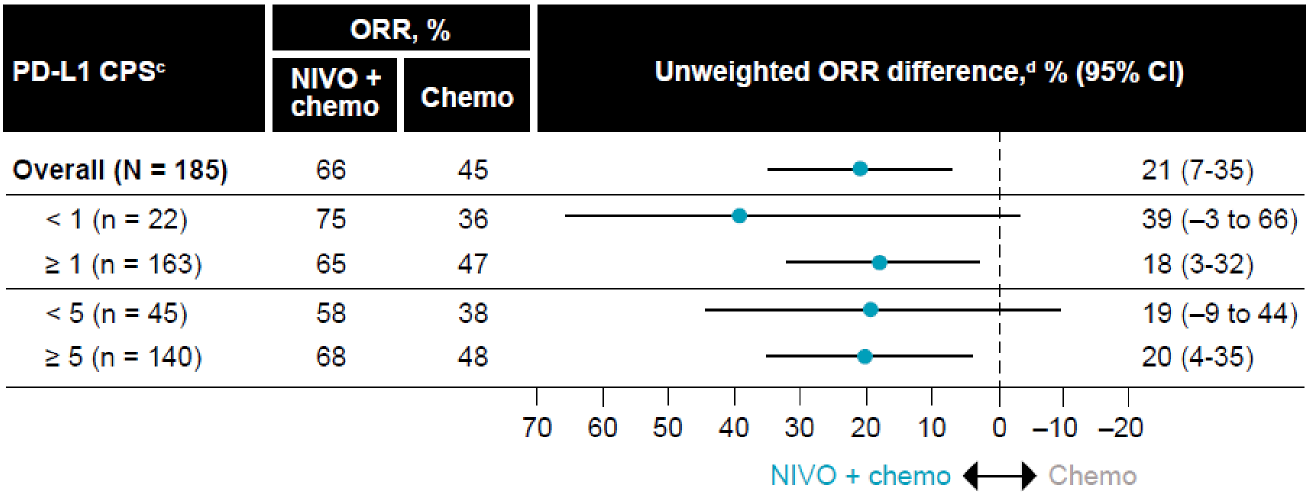

Abbreviations: CI, confidence interval; CPS, combined positive score; HR, hazard ratio; ORR, objective response rate; PD-L1, programmed death ligand 1.

[a]PD-L1 CPS expression available for all Chinese patients.

[b]Unstratified HR for death (OS) or unstratified HR for progression or death (PFS).

[c]Randomized patients who had target lesion measurements at baseline, per BICR assessment.

[d]Percentages may not reflect an exact difference due to rounding.
